# Sarcopenia – the need for an Asian perspective

**DOI:** 10.1007/s40520-025-03174-6

**Published:** 2025-08-29

**Authors:** Jean Woo

**Affiliations:** Department of Medicine & Therapeutics, Faculty of Medicine, Prince of Wales Hospital, The Chinese University of Hong Kong, Shatin, N.T., Hong Kong

**Keywords:** Sarcopenia, Community screening, Management, Social determinants, Ethnicity

## Abstract

Sarcopenia research started in Asia about 25 years ago, and is now a rapidly developing field of research. The Asian Working Group in Sarcopenia was formed in 2014 with the publication of the first Consensus definition, followed and an update in 2019, with a most recent update in 2025. Cut off values used in the definition are different from those in Europe or the US, possibly due to a combination of social determinants of body composition and ethnicity. The importance of detection of sarcopenia in community and hospital settings is recognized; however a gap exists between research, public health and clinical practice in terms of case finding and management.

The diagnosis of sarcopenia has an approximately 30 year history, first introduced in the US to describe loss of muscle mass with aging [1], followed by many epidemiological studies documenting community prevalence, risk factors, and health consequences that are independent of ethnicity, age, morbidity, income or health behavior [2, 3]. With the assignment of an ICD code M62.84 in 2016 [4], sarcopenia has been firmly classified as a disease, leading to various drug trials. Sarcopenia was initially defined as a reduction in muscle mass of two standard deviations or more below the normal mean values for younger populations. Bone densitometer was used. Using this method, the first study conducted in Asians found that the prevalence among Chinese men and women aged 70 years and over was lower compared with US data, being 12.3% in men and 7.6% in women [5]. However, the mean values for the younger population require the measurement of muscle mass of people of a different generation, which may be influenced by age-period cohort differences in socioeconomic development, nutrition, lifestyle, and the physical living environment. For example, older Chinese women were exposed to greater physical demands during their life time compared with younger women. This aspect particularly relevant given the current concepts of a life course approach to muscle health [6].

Since then, there has been a rapid increase in research into all aspects of sarcopenia in Asian countries, initially in Japan, Taiwan, China, followed by Korea and other South East Asian countries, with the formation of the Asian Working Group on Sarcopenia (AWGS) and the first consensus Asian group definition of sarcopenia. While the Asian consensus are similar to those in Europe and the US, the difference lies in the cut off values used as a criterion for normality [7, 8]. The conclusion was that this was not surprising given the ethnic differences in body shapes and sizes between Asians and Caucasians [9]. Since body composition is commonly assessed using bioimpedance, cut off values for both bioimpedance and dual-energy X ray absorptiometry were provided. Prospective studies using an outcome data driven approach, similar to that proposed by the Foundation for the National Institutes of Health [FNIH], contributed to the determination of cut off values [10, 11]. An attempt using this method was also applied to the entity of sarcopenic obesity [12]. An insight derived from these studies is that cut off values may differ for different outcomes.

With evolution of the definition of sarcopenia, inclusion of strength and physical performance measures have been included in both cross sectional and prospective cohort data in Asia. Analysis of prospective cohorts allow characterization of age-associated decline in muscle mass, grip strength and walking speed, giving some insights into variations among different ethnic groups [13]. To accompany regular updates of the AWGS consensus panel, there have been a series of studies relating to reference ranges, some including mean values in younger adults as reference [14], or comparison of values in cohorts of older adults in different Asian countries [15, 16]. A recent study pooled cross sectional data of 34,265 people from eight cohorts from age 20 onwards in Japan, Malaysia and Taiwan provided mean values and centile curves for various muscle health metrics, documenting the differing age at which decline occurs [6]. In line with the international research community, the role of nutrition in muscle health of community-dwelling adults have also been addressed by the AWGS group [17]. The close relationship between bone and muscle health had also been documented [18, 19], supporting the evolution of the current entity of osteosarcopenia [20].

Researchers in Asia also participated in various consensus groups relating to the definition [21, 22] and outcomes of sarcopenia [23]; as well as various practice guidelines [24, 25].

While there is overall consensus relating to the conceptual definition, outcomes and management of sarcopenia, the issue of reference ranges and cut off values remains controversial. These issues have not been explicitly debated: should one use population derived centile values for specific age and sex group, or relate values to young adult values, or use a data driven approach where cut off values are related to adverse outcomes in prospective studies? It is generally agreed that the latter approach may be difficult to achieve as these values need to be established by large scale population cohort studies. The former approach may be more pragmatic; however social determinants may have a significant contribution to reference ranges derived from different economies and geographic regions.

Other than social determinants, ethnic differences in body shape, size and composition also need to be taken into account, such that reference ranges may need to be developed in the context of ethnicity, geography, and other social determinants. Such observations may have impact on arriving at a universal definition of sarcopenia that includes fixed values of cut off points for muscle mass and muscle strength measurements, as well as cut off points for outcome measures. Recommendations from Consensus Panel definitions would need to take this point into account, and perhaps determine cut-off points for a particular population.

It may be argued that a universal definition with fixed cut points may not be necessary from the perspective of clinical practice. Although there is little controversy in the management of sarcopenia, a substantial research clinical practice gap remains in Asian countries. The awareness of sarcopenia is greater in the community or primary care setting. For example, in Thailand, a community screening program was recently started, carried out in villages by trained volunteers, as part of the implementation of the WHO Integrated care of older people [ICOPE] program [26]. The screening algorithm include measurement of calf circumference as well as grip strength among the first steps [27]. In Hong Kong, community screening has been piloted among members attending community centres run by non-government organizations, where sarcopenia is part of the assessment using SARC-F. If positive, links are provided to you tube educational material, and to nutrition and exercise programs https://www.cadenza.hk/e-tools/zh/ihealthscreen/. A mobile rapid geriatric assessment app that includes SARC-F has also been developed in Singapore and suitable for use in the primary care setting [28].

In the hospital setting, sarcopenia may accompany many diseases and less likely to be the focus of management, except for the non-acute rehabilitation setting or pre-habilitation programs for procedures such as knee replacement, where muscle function as well as nutrition are likely to play an important role. In the outpatient setting, again the focus is likely to be dominated by the specialty. For example, even though research shows that sarcopenia is common among patients with diabetes mellitus, screening for sarcopenia is seldom included as part of diabetes management. Nevertheless, the importance of identifying sarcopenia as part of the management of chronic diseases has been acknowledged to varying degrees among specialties. Fung et al. drew attention to the importance of diagnosis and management of sarcopenia in connection with heart failure [29]. Conversely community screening for sarcopenia using SARC-F showed a higher prevalence of older people with heart failure with preserved ejection fraction [30]. Other diseases where muscle wasting occurs include chronic obstructive pulmonary diseases, and cancer. With all these diseases, muscle wasting may be viewed as a continuum from sarcopenia to cachexia, complicating management planning.

To a certain extent, identification, standardized diagnosis and treatment depend on the health system of individual countries. For example, in the People’s Republic of China, health care is based in different types of hospitals. A group of experts convened to develop a consensus on recommendations for diagnosis and treatment of sarcopenia based in hospital clinics [31]. Nine recommendations were made, including specific recommendations for sarcopenia co-existing with diabetes mellitus, chronic obstructive pulmonary disease, chronic heart failure, and chronic kidney disease. Recommendations including regular follow-up, case conferences, conditions for referral from community to general hospitals were also included.

With population aging rapidly in many Asian countries, in spite of the emphasis on family support, residential care homes remain a necessary option for long term care. There is little research on this population of older adults. About 15 years ago, among the developed economies in Asia, Hong Kong has the highest institutionalization rate at 6.8%, followed by Japan at 3.0%. Singapore at 2.3%, Taiwan at 2.0% and China at 1.0% [32]. A profile of the institutionalized population showed high prevalence of dependencies [33]. It would not be surprising to find a high prevalence of sarcopenia. However staff numbers and quality of training are barely sufficient to provide basic quality care. The diagnosis of sarcopenia and treatment are likely to encounter practical difficulties.

A survey was carried out by the AWGS group to investigate clinical practice among healthcare professionals in Asia. While awareness of sarcopenia was high (over 90%), the percentage of respondents who screened for, diagnose, and treat sarcopenia varied from 40 to 80%, with geriatricians representing the latter group, the highest compared with other doctors, followed by allied health professionals [34]. Screening for, diagnosis and management should form a continuum, either at the same service site or be part of a robust referral infrastructure. Currently in the absence of drug therapy, the main management consists of resistance exercises and optimizing nutritional status, which is likely to be feasible over period of months in a community setting rather than in hospitals. Close links should be formed between hospital rehabilitation programs and such community facilities, if patients are being discharged from hospital with sarcopenia and some chronic disease(s). It is appropriate that geriatricians take a leading role in establishing and running such community centres, working with primary care service providers. In some Asian countries such as Japan, Taiwan, and Singapore, the government has policies encouraging such programmes, in connection with frailty. Sarcopenia screening and management are easily fitted into such models [35–37].

Thus far the majority of research have been carried out by geriatricians and gerontologists. The research clinical practice gap cannot be closed by geriatricians alone, but all health care professionals who look after older adults need to be involved. The situation may be analogous to care of the dying: not being just the responsibility of palliative care professionals, but all need to be involved. Geriatricians have a key role in raising awareness of the clinical significance of sarcopenia and participate in promoting screening, diagnosis and management in collaboration with specialists in various chronic diseases, as well as primary care physicians. Actual models of practice will depend on the health and social care systems of individual Asian countries, but geriatricians will need to play a leading role.

In conclusion, this article traced the development of sarcopenia research in Asia shortly after the concept of age-related muscle loss was introduced, the bulk of which took place in the community. Pooled data enabled consensus panel diagnostic criteria for Asia based on available evidence from prospective cohort studies. However, the influence of ethnicity and social determinants may represent challenges in the cut off values use to operationalize the diagnosis of sarcopenia. These may need to be established on a regional basis. From a pragmatic point of view, community case finding has found increasing support, while there has been a slower uptake in hospitals and residential care homes for the elderly.

## Data Availability

No datasets were generated or analysed during the current study.
